# Editorial: Teaching and learning in higher education: the role of emotion and cognition

**DOI:** 10.3389/fpsyg.2023.1230472

**Published:** 2023-07-04

**Authors:** Jian-Hong Ye, Mei-Yen Chen, Yung-Wei Hao

**Affiliations:** ^1^Faculty of Education, Beijing Normal University, Beijing, China; ^2^National Institute of Vocational Education, Beijing Normal University, Beijing, China; ^3^Graduate Institute of Sport, Leisure and Hospitality Management, National Taiwan Normal University, Taipei City, Taiwan; ^4^Graduate Institute of Curriculum and Instruction, National Taiwan Normal University, Taipei City, Taiwan

**Keywords:** cognition, competencies, core literacy, emotion, higher education, learning, teaching

With the global boom in higher education and the wave of educational reforms, the promotion of “meaningful teaching and learning” has become an important topic of discussion and has attracted the attention of many international scholars. This is because the provision of quality education is one of the United Nations' sustainable development goals. Therefore, for the purpose of quality education development, research from a psychological perspective has become a mainstream research direction in the field of teaching and learning. This is because a psychological perspective can help teachers in higher education better understand their teaching performance from a micro perspective, and can enhance or promote higher quality learning performance and effectiveness, thereby equipping students with the core literacy and competencies required for the 21st century.

There are many worthwhile references to emotion and cognition Research Topics within the topic of teaching and learning in higher education, including: academic achievement (e.g., Liu et al.), academic persistence (e.g., Ma et al.), academic success (e.g., Pishghadam et al.), anger (e.g., Deng et al.), anxiety (e.g., Ma; Qiu and Luo), autonomy support (e.g., Zhang et al.), beliefs (e.g., Huang et al.; Qian et al.), career adaptability (e.g., Lu and Jia), career exploration (e.g., Lu and Jia), conscientiousness (e.g., Huang et al.), continuance intention (e.g., Li X. et al.), critical thinking (e.g., Cheng; Li M. et al.), emotion (e.g., Bachler et al.; Maier et al.; Qin et al.; Schlosser and Paetsch), emotion regulation (e.g., Cheng; Deng et al.; Li M. et al.; Zheng et al.; Weidi and JeeChing), emotional intelligence (e.g., Jiang et al.), engagement (e.g., Al-Obaydi et al.; Deng et al.; Ma et al.; Wang et al.; Zhao and Ling), enjoyment (e.g., Zhang et al.), evaluation of teaching (e.g., Keerthigha and Singh; Zhao et al.), grit (e.g., Zheng et al.), intelligence (e.g., Pishghadam et al.), interests (e.g., Bi and Liu), learning strategies (e.g., Alshaharni), life satisfaction (e.g., Weidi and JeeChing), motivation (e.g., Bi and Liu; Keerthigha and Singh; Zhao and Ling), occupational intention (e.g., Zhang), performance (e.g., Cheng; Qiu and Luo), professional success (e.g., Yang), psychological needs (e.g., De Vocht et al.), reflection (e.g., Schlosser and Paetsch), resilience (e.g., Wu), self-efficacy (e.g., Deng et al.; Liu et al.; Ma; Schlosser and Paetsch; Zheng et al.), self-management (e.g., Zhao and Ling), self-monitoring (e.g., Zhao and Ling), self-regulated learning (e.g., Bellhäuser et al.), stress (e.g., Drüge et al.; Zhang), teacher aggression (e.g., Yang), teacher burnout (e.g., Yang; Zhang), teacher support (e.g., Ma et al.), teaching preparedness (e.g., Huang et al.), teaching styles (e.g. Keerthigha and Singh), values (e.g., Bi and Liu), and wellbeing (e.g., Zhao and Ling). It can be seen from the above studies that the variables of research on teaching and learning are quite diverse.

Although there have been many studies that have confirmed the importance of emotion and cognition in higher education, with the passage of time, rapid technological developments, literacy-oriented philosophies, and changes in teaching philosophies during the COVID-19 pandemic, many of the originally known results may have changed due to different circumstances. For example, although research on online learning in higher education has been available for decades, there has been a very large amount of research on online learning or online teaching in the past few years due to the COVID-19 pandemic, which led to the temporary closure of classrooms. However, these findings seem to differ in many ways from previous studies. Prior to the pandemic, many studies found that online learning worked well for learners.

The implementation of teaching and learning in emergency situations seems to have difficulty achieving the same good results as deliberately created teaching and learning situations, and can even lead to poor learning for learners. This is because many previous studies excluded disruptive factors, but these disruptive factors are actually present in the classroom. While empirical studies have been successful in proving the validity of the new approach, the scenarios are not realistic. It is important that these excluded distractors are also evaluated after the validity of the method has been confirmed, in order to gain a more complete understanding of the effectiveness of the new method in real educational settings. Although COVID-19 regulations are gradually being relaxed around the world, and teaching is returning to offline classrooms, the impact of the COVID-19 epidemic on education remains, as the experience of teaching during the epidemic has given us insight into the practical effects of current online learning and the potential problems that may exist. This will also help to improve educational technology and digital learning methods in the post-epidemic era, and to explore how to enhance learning to compensate for the loss of learning during the epidemic, as well as how to better design blended learning, collaborative learning, online learning, self-directed Learning, self-regulated Learning, personalized learning, flipped classrooms, and so on.

Meanwhile, under the digital transformation generation of education, metaverse technologies such as 5G, cloud computing, artificial intelligence, artificial intelligence generated content (e.g., ChatGPT), augmented reality (AR), virtual reality (VR), mixed reality (MR), extended reality (XR), substitutional reality (SR), holographic displays, blockchain, IoT, and human-computer interaction are considered to help facilitate quality (meaningful) teaching and learning. Moreover, due to the increasing maturity of AI technology, its role in the field of education has became more important. AI is may be used to assist teachers and students in academic assessment, consultation, and guidance through AI-based educational technologies, enabling personalized learning. It can even be used for chat and psychological counseling through its conversational capabilities. However, research on these topics still needs to continue, as most of the metaverse technologies are not yet widespread in the classroom or are not yet well-adapted for use in the classroom. There is a need for more research that combines educational experience with user experience, so that educators can understand how to use these technologies more effectively and avoid poor use, and so that teaching and learning can be more diverse, personalized and contextualized. The basis of this type of research is not just about effectiveness, but also about the user's sense of experience (adaptability).

It is important to understand how learning takes place. People always want to replicate successful experiences, but how to avoid failure also needs to be taken into account. Success is difficult to replicate in its entirety because of individual differences, but the same mistakes should be avoided as much as possible. Moreover, even students of the same level of learning may encounter different problems when studying the same content, let alone learners from different subjects and systems. We therefore urge educators and researchers to understand the difficulties and needs of learners, and to collect more complete information on problems in different contexts and situations, so that they can provide appropriate help to students. At the same time, the framework, standards and objectives of curriculum and teaching may vary according to the educational policies of different countries and regions. For example, China advocates the inclusion of moral concepts in the curriculum to nurture talents (known as curriculum ideology and politics), which has a Chinese effect on students' cognition, emotions, skills and knowledge. Such a curriculum model and its effects will have national, ethnic and regional characteristics and should therefore be of interest to academics as well, depending on the educational policy context.

In addition, although education experts, the government and educational organizations advocate literacy-oriented education and student-centered teaching methods, traditional didactic teaching is still the mainstream teaching method. Therefore, how to create a truly effective educational environment (learning context) is still an important issue. It is also important that students become self-directed learners, and that teachers become facilitators, guides and companions. Of course, not all teaching and curriculum designs are effective. In particular, design approaches that are not grounded in theory are less likely to deliver effective results. Moreover, it is not only theoretical foundations that are needed for the design of teaching and curricula. The evaluation of learning outcomes also requires a theoretical basis. In addition to identifying the effectiveness of teaching and learning implementation, it is important to identify the pre- and post-teaching relationships that influence teaching and learning. This is because these factors are thought to influence learners' beliefs, intentions, wishes, behaviors (actions) and outcomes. This is why it is important to understand the emotional and cognitive factors of the students (the learners). Therefore, teaching and learning should not only focus on changes in knowledge and skills, but also on cognitive and emotional responses. In addition, among the issues of teaching and learning in higher education, there is still much to explore in the areas of education policy-based practices, sustainable development, localization of international education goals, curriculum development, instructional design, STEAM education (C-STEAM, iSTEAM, IP-STEAM, etc.), educational technology applications, online learning, innovative learning strategies, learner-centered learning/teaching (mixed methods, personalisation, inquiry-oriented, work-oriented, competition-oriented, context-oriented, collaboration-oriented, topic-oriented, game-oriented, problem-oriented, literacy-oriented), beliefs about teaching and learning, teacher professional capacity development, and teaching adaptability.

Moreover, the models proposed by learning theories are not static. Teachers need to adapt the theoretical models more effectively according to student characteristics, subject matter, curriculum content, course context and lesson time. Therefore, the flexibility and variability of teaching methods need to be taken into account, and the validity of new models needs to be tested. In addition, future research on teaching and learning will need to emphasize the implementation of experiments in real-world learning contexts. At the same time, the ethics of research will increasingly focus on the protection of students' rights when participating in experiments. A single-group experimental design would be an appropriate approach to research design for the benefit of all students, given the constraints of insufficient numbers of participants and the difficulty of re-grouping experiments. After all, we have already recognized the limitations or shortcomings of traditional lecture-based teaching and learning, which is why we want to try out new teaching (learning) methods.

It is also important that innovative (learning and teaching) methods are applied in a sustainable manner. If an effective educational technique, teaching method or learning method was developed but was then not applied after the completion of the teaching experiment, the end of the research project or the publication of a journal paper, it would be a shame and a waste of educational resources. In addition, although the indicators can be converted into numerical values for assessment and comparison, this does not cover all of the assessment content. There is much that needs to be understood through qualitative content. By combining the results of these two areas, a more comprehensive picture of the truth can be obtained. We therefore call for a more holistic approach to the study of teaching and learning.

## Author contributions

All authors read and approved the final manuscript.

